# Effects of nitrate and ammonium on assimilation of nitric oxide by *Heterosigma akashiwo*

**DOI:** 10.1038/s41598-023-27692-3

**Published:** 2023-01-12

**Authors:** Emily M. Healey, Stacie Flood, Patience K. Bock, Robinson W. Fulweiler, Joanna K. York, Kathryn J. Coyne

**Affiliations:** 1 College of Earth, Ocean, and Environment, University of Delaware, Lewes, 19958 USA; 2 Mote Marine Laboratory, Sarasota, 34236 USA; 3 Department of Earth & Environment and Department of Biology, Boston University, Boston, 02215 USA; 4 Present Address: Maryland Institute for Applied Environmental Health, University of Maryland, College Park, 20742 USA; 5 Present Address: South Florida Water Management District, Coastal Ecosystems Section, West Palm Beach, FL 33406 USA

**Keywords:** Biochemistry, Marine biology

## Abstract

The harmful alga *Heterosigma akashiwo* possesses a hybrid nitrate reductase (NR) enzyme, NR2-2/2HbN, which has the potential to convert NO to nitrate for assimilation into biomass. In previous research, *NR* transcription in *H. akashiwo* was induced by nitrate while NR activity was inhibited by ammonium. Here, the capacity of *H. akashiwo* to use NO in the presence of nitrate and/or ammonium was investigated to understand the regulation of NO assimilation. Continuous cultures of *H. akashiwo* were acclimated to growth on nitrate, ammonium, or a mixture of both. Aliquots from these cultures were spiked with ^15^N-labeled NO. The expression of genes involved in nitrogen assimilation was evaluated, as well as nitrate reductase activity and assimilation of ^15^N-labeled nitrogen into algal biomass. Results showed that NO induced expression and activity of NR, and upregulated expression of *GOGAT* regardless of the presence of other inorganic nitrogen sources, while *GS* expression decreased over time. Furthermore, ^15^NO uptake and assimilation was significantly higher in cultures acclimated for growth on ammonium compared to cultures acclimated for growth on nitrate alone. Assimilation of NO may provide *H. akashiwo* with a competitive advantage in N-poor environments or areas with elevated NO.

## Introduction

Harmful algal blooms (HABs) are widespread coastal phenomena that occur when accumulation of algal species have negative socioeconomic, public health or environmental impacts^1^. Among HABs, those caused by the globally distributed toxic species, *Heterosigma akashiwo* (Raphidophyceae) pose a significant threat to the global finfish and shellfish economy, with documented losses reaching into the millions of dollars^2^. Understanding environmental drivers that contribute to the proliferation of *H. akashiwo* is essential to preventing and mitigating blooms of this species.

A major factor in HAB formation is the availability of nitrogen both in terms of concentration and type^3,4^. Indeed, past research showed that *H. akashiwo* removes a substantial portion of nitrogen from coastal waters during blooms^5^, suggesting that nitrogen plays a role in *H. akashiwo* bloom initiation and maintenance. Although *H. akashiwo* can use both nitrate and ammonium^6–8^, there is evidence that *H. akashiwo* can also use nitric oxide (NO) and maintain positive growth with NO as a sole nitrogen source^9–11^. As a reactive nitrogen species, NO is involved as a signaling molecule in numerous plant and animal functions, including pathology and stress response^12^. NO also reacts with transition metals and proteins^13^ and is not considered a viable nitrogen source for eukaryotes due to its toxicity. However, concentrations of NO approaching 500 nM, with spikes exceeding 200 µM, have been documented at the oxic-anoxic transition zone of coastal marine sediments^14,15^ where it is produced as an intermediate in heterotrophic denitrification^16^. Consequently, NO may be accessible to *H. akashiwo* as it undergoes diel vertical migration^17^.

The potential for NO assimilation by *H. akashiwo* may be due to a unique hybrid nitrate reductase, NR2-2/2HbN (NR2) found in this species and at least one other raphidophyte^9^. Nitrate reductase (NR) catalyzes the rate-limiting reduction of nitrate to nitrite, with electrons provided by NAD(P)H^18^. Nitrite is transported into the chloroplast, where it is reduced to ammonium by nitrite reductase (NiR). Ammonium is then incorporated into amino acids through the glutamine synthetase/glutamine:2-oxoglutarate aminotransferase (GS/GOGAT) pathway. NR is highly regulated in plants and algae, both transcriptionally and post-transcriptionally, in part by the presence and relative proportions of nitrate and ammonium^18–22^. In some plants and algae, NR transcription and activity is upregulated by nitrate and repressed by nitrite and ammonium as well as downstream products of the GS/GOGAT pathway^22^.

In contrast to prototypical NR, however, NR2 in *H. akashiwo* includes a truncated hemoglobin (HbN) domain^9^. Similarities to mycobacterial HbN^23^ and truncated hemoglobins in other algal species^24^ suggest that NR2 converts NO to nitrate via nitric oxide dioxygenase (NOD) activity of the HbN domain^9^, which may then be reduced to nitrite via NR activity. In support of this hypothesis, NR in *Chlamydomonas* has been shown to associate with truncated hemoglobin (THB1) to convert NO to nitrate^24^. However, NR also participates in generation of NO in *Chlamydomonas* through partnership with amidoxime-reducing component/nitric oxide-forming nitrite reductase (ARC/NOFNiR)^25^. The generation of NO through this pathway plays a role in repression of NR by ammonium, and has been shown to repress *NR* transcription and enzyme activity as well as nitrate transport when both ammonium and nitrate are present^26,27^. Assimilation of externally sourced NO through NOD activity of NR2 in *H. akashiwo* may be inhibited in a similar manner, effectively preventing *H. akashiwo* from accessing this nutrient when ammonium is present.

The object of this study was to investigate the regulation of NO assimilation into biomass by *H. akashiwo*, and the effects of other nitrogen sources on this process. Specifically, this study tested the hypothesis that the assimilation of NO is repressed in the presence of ammonium. This may affect the ability of *H. akashiwo* to access this nitrogen source in coastal environments impacted by ammonium pollution, and limit the role of NO in bloom initiation and maintenance.

## Results

**Figure 1:**
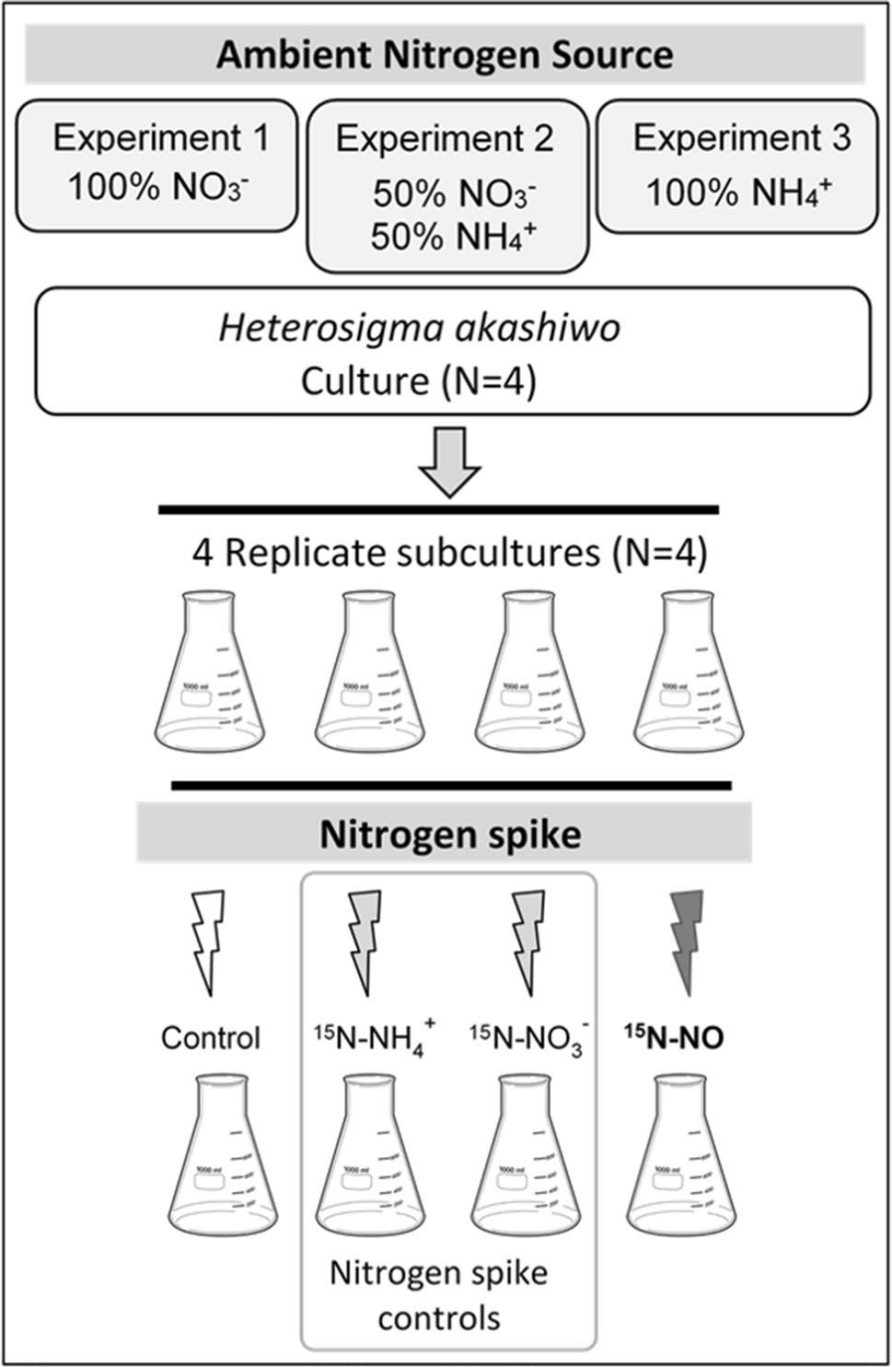
Experimental design. *Heterosigma akashiwo* was cultured with 100 µM nitrate (Experiment 1), 50 µM nitrate and 50 µM ammonium (Experiment 2), or 100 µM ammonium. Cultures (N = 4) were then subdivided into four treatments for a total of 16 subcultures for each experiment. Controls received nitrogen free medium. Nitrogen-spiked controls received ^15^N-labelled nitrate or ammonium at 10% of ambient concentrations (see Table 1), and the NO treatment was spiked with 100 µM ^15^N-labelled S-nitroso-N-acetylpenicillamine (SNAP) to generate ^15^N–NO.

For this investigation, *Heterosigma akashiwo* was acclimated to growth on nitrate (Experiment 1), a 50/50 mix of nitrate and ammonium (Experiment 2) or ammonium alone (Experiment 3) (see Fig. 1). Subcultures from each experiment were spiked with ^15^NO. Several parameters were measured to evaluate the response of *H. akashiwo* to addition of NO, including transcription of genes involved in nitrogen assimilation, NR enzyme activity, and assimilation of ^15^N-labelled NO into particulate nitrogen. Paired replicates also received either nitrate or ammonium at 10% ambient concentrations instead of NO as a means to verify responses by *H. akashiwo* to commonly encountered nitrogen sources.

### Gene expression

**Figure 2:**
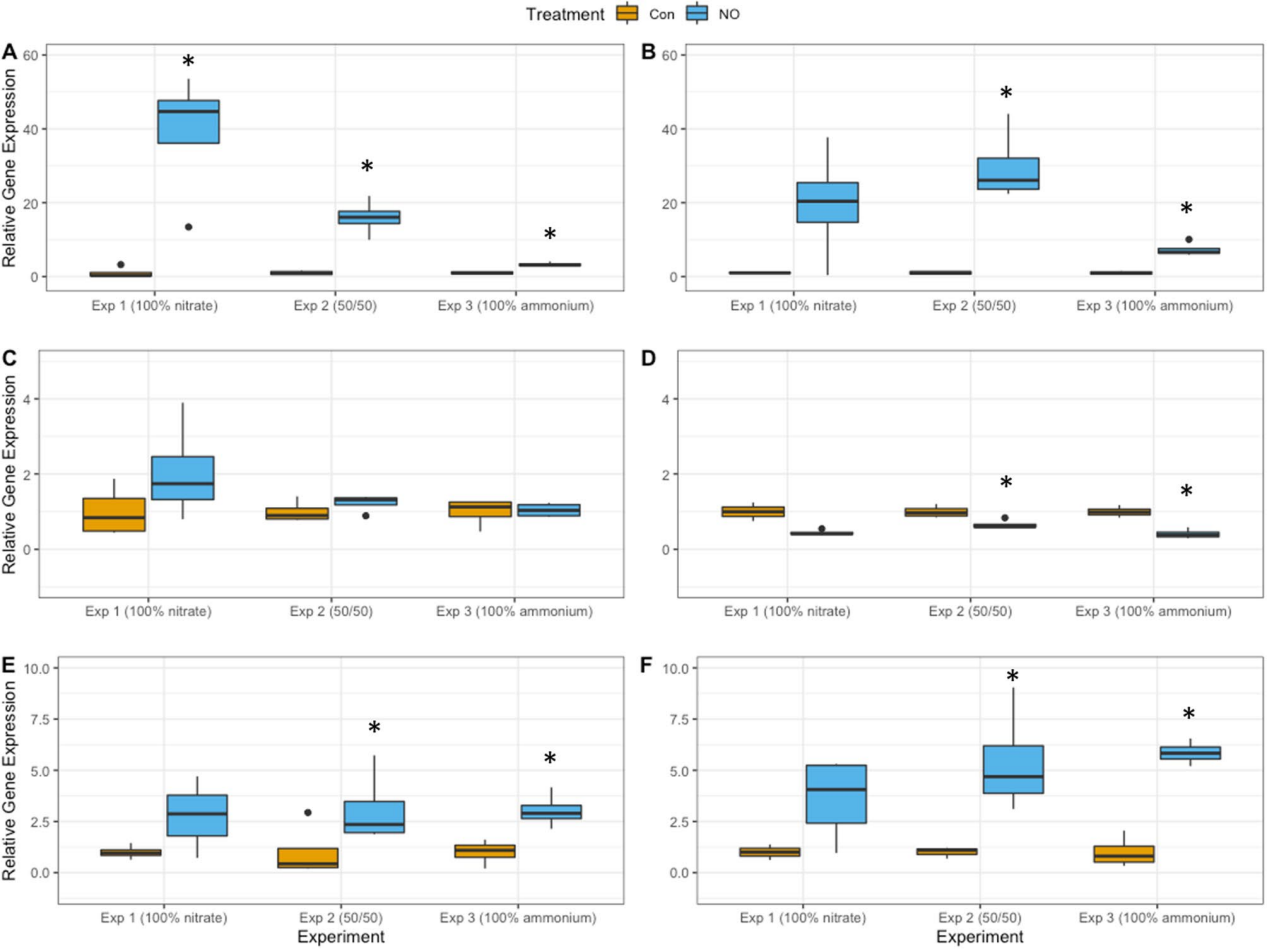
Gene expression analysis of *Heterosigma akashiwo* after spiking with NO. Relative gene expression of nitrate reductase (*NR*; **A**, **B**), glutamine synthetase (*GS*; **C**, **D**) and glutamine:2-oxoglutarate aminotransferase (*GOGAT*; **E**, **F**) at 15 min (**A**, **C**, **E**) and at 60 min (**B**, **D**, **F**) after spiking with NO for control and treatment cultures of *Heterosigma akashiwo* when acclimated to growth on 100 µM nitrate (Exp 1), 50 µM nitrate and 50 µM ammonium (Exp 2), or 100 µM ammonium (Exp 3). Expression was normalized to *GAP* transcript abundance. Error bars are + /− 1 standard deviation from the mean. Significant difference in relative expression of genes in the nitric oxide treatment compared to controls (*) is denoted.

The expression of *NR*, *GS*, and *GOGAT* was normalized to the transcript abundance of *GAP* (Figs. 2 and S1). *NR* expression in the NO-spiked samples was significantly higher than the Control for each experiment at 15 min (Fig. 2A). Mean *NR* expression was highest in Experiment 1, and lowest in Experiment 3. At 60 min, the relative expression of *NR* remained significantly higher than Controls for Experiments 2 and 3 (Fig. 2B), but there were no significant differences between Control and NO treatment for Experiment 1 at 60 min. The large standard deviation between biological replicates at this time point was likely due to the highly dynamic gene expression activity across biological replicates in Experiment 1. A comparison between time points indicates that the expression of *NR* decreased over time for Experiment 1 and increased over time for Experiments 2 and 3.

*GS* expression in the NO-spiked samples was not significantly different than the Controls for any experiment at 15 min, but was significantly lower than the Controls in Experiments 2 and 3 at 60 min (Fig. 2C, D). Similar to *NR* expression, *GS* expression was the highest in Experiment 1, and lowest in Experiment 3 at 15 min, while at 60 min, the mean *GS* expression was highest in Experiment 2 and lowest in Experiment 3. A comparison between time points indicates that the expression of *GS* relative to the Control decreased over time for all three experiments.

*GOGAT* expression in the NO-spiked samples at each time point was significantly higher than the Control in Experiments 2 and 3, but not significantly different from the Control in Experiment 1 (Fig. 2E, F). *GOGAT* expression in NO treatments at 15 min was similar across all three experiments. At 60 min, the mean expression of *GOGAT* was highest in Experiment 3 and lowest in Experiment 1. A comparison between time points indicates that the expression of *GOGAT* relative to Controls increased over time for all three Experiments (Fig. 2E, F).

Expression of *NR, GS* and *GOGAT* in nitrate- and ammonium-spiked treatments are shown in Supplementary Fig. S1 (available online). The amount of added nitrate and ammonium (10% of ambient) differed by treatment and by experiment, so that direct comparisons should be viewed with caution. Instead, the nitrate and ammonium treatments provided a qualitative assessment of *H. akashiwo*’s response to more commonly encountered nitrogen sources, and to provide context for its response to NO addition. *NR* and *GS* expression patterns and magnitude of expression relative to Controls when spiked with nitrate at both 15 and 60 min were similar to those of NO treatments. As expected, addition of ammonium inhibited *NR* expression relative to Controls at 15 min for all three experiments, while *GS* expression patterns when spiked with ammonium were similar to those of NO treatments at both time points. *GOGAT* expression at 15 min also followed the pattern of response observed for NO treatments. At 60 min, however, *GOGAT* expression for nitrate-spiked samples was lowest in Experiment 3, and lowest in Experiment 1 when spiked with ammonium.

### Nitrate reductase enzyme assay

**Figure 3:**
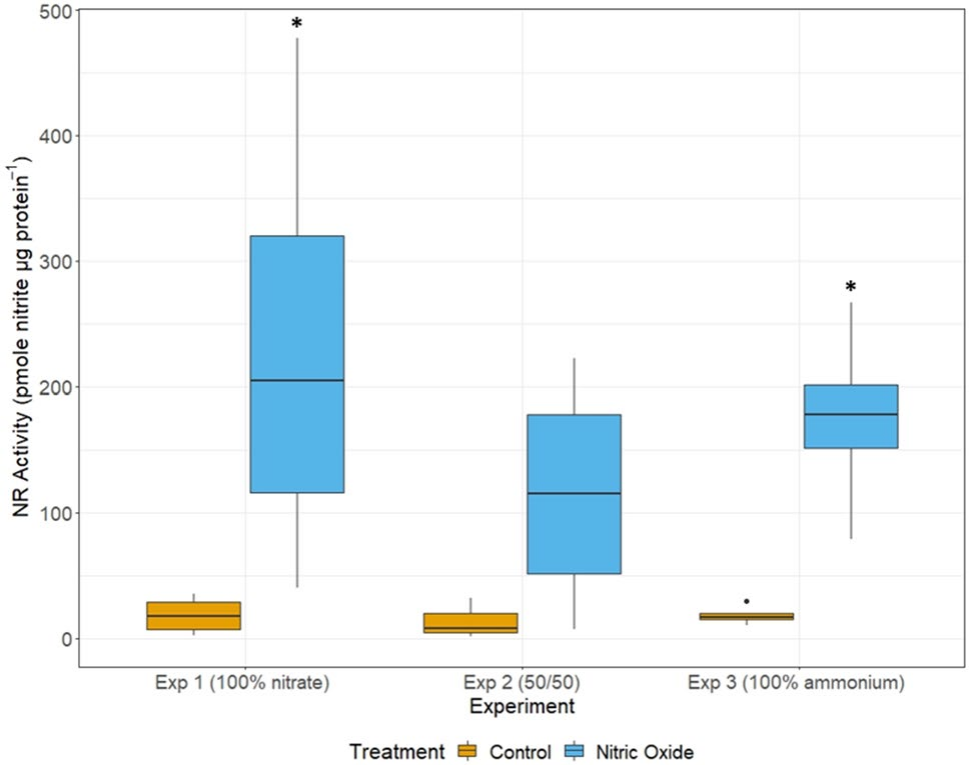
Nitrate reductase enzyme activity in *Heterosigma akashiwo* after spiking with NO. Nitrate reductase (NR) enzyme activity 2 h after spiking with NO for Control and treatment *Heterosigma akashiwo* cultures when acclimated to growth on 100 µM nitrate (Exp 1), 50 µM nitrate and 50 µM ammonium (Exp 2), or 100 µM ammonium (Exp 3). Expression was normalized to protein content. Error bars are + /− 1 standard deviation from the mean. Significant difference in activity in the nitric oxide treatment compared to controls (*) is denoted.

NR activity, measured at 2 h after spiking with NO, was up to 12 times higher than Controls, with no significant differences between NR activity between experiments (Fig. 3). NR activity was significantly higher than Controls in Experiments 1 and 3, but no significant differences were detected between Control and NO treatment in Experiment 2.

As with the gene expression analysis, direct comparisons between treatments or between experiments were not possible due to the differences in nitrogen added (10% ambient) for ammonium and nitrate. However, NR activity for nitrate- and ammonium-spiked cultures followed a pattern similar to NO treatments with highest activity in Experiment 1 and lowest activity in Experiment 2 (see Supplementary Fig. S2 online).

### Nitrogen assimilation (^15^N)

**Figure 4:**
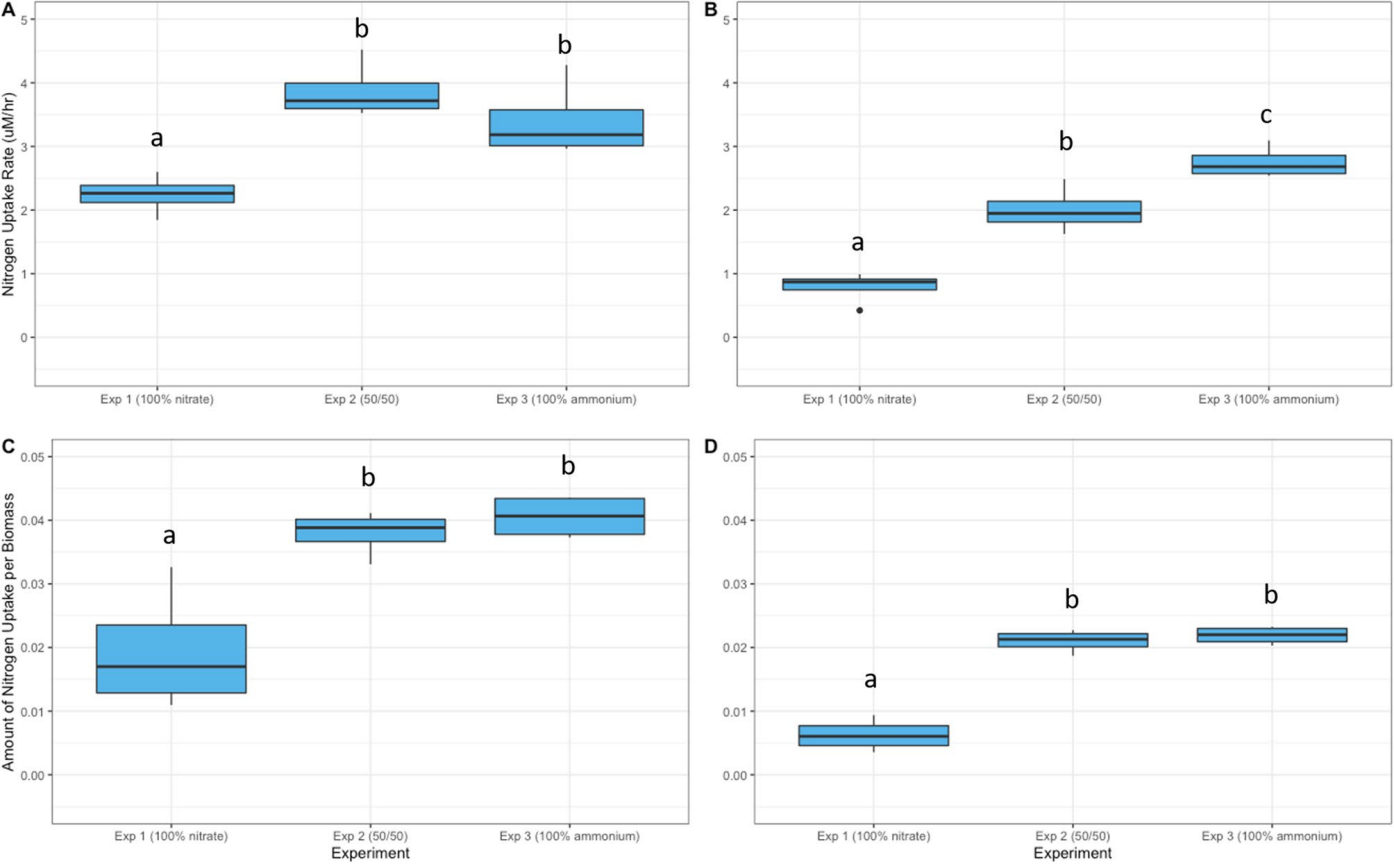
Assimilation of NO for *Heterosigma akashiwo*. Nitrogen uptake rate (**A**, **B**) and amount of nitrogen uptake per biomass (**C**, **D**) at 4 h (**A**, **C**) and 24 h (**B**, **D**) after spiking with ^15^NO for *Heterosigma akashiwo* cultures when acclimated to growth on 100 µM nitrate (Exp 1), 50 µM nitrate and 50 µM ammonium (Exp 2), or 100 µM ammonium (Exp 3). Error bars are + /− 1 standard deviation from the mean. Significant difference in the ^15^N-nitric oxide uptake rate and amount of ^15^N-nitric oxide uptake per biomass between experiments is denoted (a, b, c).

^15^N uptake and assimilation was analyzed at 4 (T4) and 24 (T24) hours after spiking with ^15^N-labeled N. At T4, the ^15^N uptake rate (Fig. 4A) of the NO-spiked samples was significantly higher in Experiments 2 and 3 compared to Experiment 1 (*p* value < 0.05). Likewise, the ^15^N uptake per biomass (Fig. 4C) for NO-spiked samples was significantly higher in Experiments 2 and 3 compared to Experiment 1 at T4 (*p* value < 0.05).

The uptake rate of ^15^N at T24 was significantly different between all three experiments (Fig. 4B). The highest ^15^N uptake rate in NO-spiked samples occurred in Experiment 3 and the lowest was in Experiment 1. ^15^N-labelled NO uptake per biomass was highest in Experiment 3 and lowest in Experiment 1 at T24 (Fig. 4D). ^15^N uptake per biomass for NO-spiked samples was not significantly different between Experiments 2 and 3 (*p* value < 0.05).

## Discussion

Stewart and Coyne^9^ described a unique enzyme, NR2-2/2HbN (NR2) in *Heterosigma akashiwo*, that includes a HbN domain, potentially coupling nitric oxide dioxygenase (NOD) with nitrate reductase (NR) activity to assimilate NO. The ability to use NO as a nitrogen source has also been demonstrated for this species^10,11^, which may provide *H. akashiwo* with an advantage over other algae in regions where NO is available. This study investigated the effects of competing nitrogen types (nitrate, ammonium or an equal mix of nitrate and ammonium) on the ability of *H. akashiwo* to use NO. Transcript abundance for key genes involved in nitrate reduction (*NR*) and ammonium assimilation into amino acids (*GS* and *GOGAT*), as well as NR activity and ^15^NO uptake rate and assimilation into biomass were measured. Overall this research demonstrated that NO stimulated transcription of *NR* and *GOGAT*, as well as NR activity, resulting in incorporation of ^15^NO into biomass by this alga.

Coyne^28^ showed that *NR* is constitutively expressed in *H. akashiwo* and upregulated by addition of nitrate. *NR* transcripts were detected under nitrogen starvation as well as in the presence of ammonium. Results presented here demonstrated that exposure to NO also increased expression of *NR* in *H. akashiwo* compared to Control cultures, regardless of the presence of other nitrogen sources (Fig. 2). Mechanisms involved in nitrogen uptake differ depending on nitrogen type: Nitrate and ammonium are moved into cells through inducible transporters^29^, while NO rapidly diffuses into cells^30^. Indeed, the highest expression of *NR* was measured at 15 min in cultures acclimated to growth on nitrate (Fig. 2A). Even when acclimated to ammonium as a sole source of nitrogen (Experiment 3), however, *NR* transcript abundance was significantly higher than Controls within 15 min after spiking with NO. Prior research showed that exposure to NO has a variable effect on *NR* transcription in plants and other algal species, with reports of increased transcript levels^31^, inhibition^26^ or no changes in transcript levels^32^. The mechanism for upregulation of *NR* expression by NO in *H. akashiwo* is not clear, but one possibility is that nitrate generated by NOD activity of constitutively expressed NR2 may have been sufficient to induce upregulation of *NR*. Comparison of NO spiked subcultures over time also supports the hypothesis that *NR* expression in response to NO is governed by regulatory mechanisms similar to cultures exposed to nitrate (see Supplementary Fig. S1 online). Interestingly, the magnitude of difference in the relative expression of *NR* in NO spiked subcultures decreased over time in cultures grown on nitrate alone (Experiment 1), indicating potential feedback inhibition from downstream products^33,34^. Glutamine, for example, has been shown to inhibit transcription of *NR* in *Nicotiana plumbaginifolia*^35^, while glutamine-derived ammonium downregulated *NR* in *Dunaliella viridis*^36^.

The increase in expression of *NR* after 60 min in NO-spiked subcultures acclimated to growth with ammonium (Experiment 3) or a mix of ammonium and nitrate (Experiment 2) may be due to alleviation of ammonium repression on transcriptional activity. In previous research, Coyne^28^ showed that *NR* transcripts increased after nitrate was added to *H. akashiwo* cultures that were grown with ammonium, suggesting that nitrate: ammonium concentrations play a role in regulating expression^28^. Likewise, nitrate produced from NO through NOD activity of NR2 may have increased intracellular nitrate: ammonium over time to overcome ammonium inhibition on *NR* transcription.

Unlike *NR*, transcription of the cytosolic form of GS examined here did not immediately respond after exposure of the culture to nitric oxide. The activity of cytosolic GS coordinates nitrogen and carbon use efficiency and plays a major role in the assimilation of inorganic nitrogen as well as nitrogen recycling within the cell^37^. In diatoms, transcripts of cytosolic *GS* increased with the onset of darkness^38^, suggesting that it may also participate in dark assimilation of nitrogen. Here, expression of cytosolic *GS* in *H. akashiwo* at 15 min after spiking with NO was not significantly different from Controls, regardless of ambient nitrate or ammonium concentrations (Fig. 2C). The subsequent decrease in *GS* transcript abundance for NO-spiked cultures at 60 min compared to Controls for all three experiments suggests feedback inhibition from products of N assimilation (Fig. 2D). While little is known about the regulation of *GS* transcription in eukaryotic algal species, results of this study are consistent with research on plants and bacteria, indicating that transcription responds to cellular nitrogen status and is downregulated in response to feedback inhibition by accumulation of organic nitrogen products within the cell.

Results of this study showed that *GOGAT* expression was upregulated by NO addition in cultures acclimated to growth on ammonium (Experiment 3) or ammonium and nitrate (Experiment 2; Fig. 2E, F). It has been shown that expression of *GOGAT* is responsive to ammonium concentrations^39,40^, so that the increase in *GOGAT* expression over time in the present study was likely due to accumulation of downstream products of nitrate reduction, including ammonium and glutamine^39^. However, there is little supporting evidence of GOGAT regulation in microalgae and additional studies are required to fully understand mechanisms involved in regulation of both *GS* and *GOGAT* expression in these species.

Gene expression provides insight on the transcriptional response to nitrogen input but assimilation of nitrogen is also controlled post-transcriptionally, through mechanisms regulating the activity of the enzymes. The effect of NO on the activities of enzymes in nitrogen assimilation pathways has been studied in only a few model species. In *Chlamydomonas reinhardtii*, NR activity as well as the uptake of ammonium and nitrate were inhibited by NO^41^. Inhibition of NR in *C. reinhardtii* was mediated by truncated hemoglobins (THBs) with homology with the HbN domain of *H. akashiwo* NR2^24^. Although NR activity in *H. akashiwo* was previously found to be inhibited by ammonium^42^, the observed increase in NR activity with addition of NO in the current study was not significantly affected by the presence of ammonium (Fig. 3). It should be noted that NOD activity of NR2 was not measured here, and NR activity was used as a proxy for dioxygenase activity of NR2, converting NO to nitrate. Overall, results of the NR assay indicate that *H. akashiwo* responds to NO as it would to nitrate^28^, suggesting that increasing NO led to an increase in nitrate: ammonium concentrations to overcome repression of NR activity by ammonium.

While not measured here, less is known about the effects of nitrogen type on GS and GOGAT enzyme activities of algal species. Regulation of GS has been detailed primarily in plants^37^ and bacteria, including cyanobacteria^43^, where a broad array of N-containing biomolecules indicative of cellular N status participate in cumulative feedback inhibition of GS activity. The study of dinoflagellate *Akashiwo sanguinea* showed that GS activity was highest when grown in ammonium, but was still active in the presence of nitrate, and steadily increased until high glutamate concentrations were reached^44^. A study of phytoplankton in the Taiwan strait showed a positive relationship between GS activity and ammonium but noted that this relationship was not maintained in unialgal culture studies of *Emiliania huxleyi* or *Dunaliella primolecta*^45^. Information on the induction and repression of GOGAT activity in eukaryotic algae is also limited. As with NR, GOGAT activity is likely to be modulated by the presence of substrates (glutamine and 2-oxoglutarate) and products. The present study indicated a trend toward increasing GOGAT expression with time after spiking with NO (Fig. 2E, F), suggesting a similar increase in substrate and GOGAT activity. An increase in GOGAT activity with increased transcription is also supported by the incorporation of ^15^NO into cellular nitrogen as discussed below (Fig. 4).

The increased incorporation of ^15^NO into biomass by cultures acclimated to growth on ammonium (Experiments 2 and 3; Fig. 4) is consistent with results reported previously for this strain of *Heterosigma*, showing a preference for nitrate over ammonium for this species^6^. In addition, the lower NO uptake rate and assimilation in the presence of nitrate (Experiment 1) compared to cultures with ammonium present may also be due to competition at the active site on NR. Nitrate binds to the molybdenum-molybdopterin cofactor, Mo-MPT, on NR^46^. Unlabeled nitrate present in the growth medium for Experiment 1 (see Fig. 1) and ^15^N-labelled nitrate produced by the conversion of ^15^NO to nitrate may compete for the active site of NR, resulting in the reduced uptake rate and lower amount of NO assimilation in the presence of nitrate compared to Experiments 2 and 3, where unlabeled nitrate was lower or absent altogether.

Diffusion may play a role in reduced uptake rate of the NO at 24 h compared to 4 h (Fig. 4). Since NO rapidly diffuses into the cell^29,30^, the abundance of NO within the cell available for assimilation would be greatest immediately after spiking cultures. As ^15^NO was depleted over time, the ambient, unlabeled nitrogen in the form of either nitrate or ammonium would continue to be transported into the cell and assimilated into biomass, effectively diluting the signal from ^15^NO.

## Conclusions

Results of this investigation do not support the hypothesis that uptake and assimilation of NO by *H. akashiwo* is inhibited by ammonium. Indeed, NO upregulated NR expression and resulted in an increase in NR activity regardless of the presence of other inorganic nitrogen sources, with an increase in ^15^NO uptake and assimilation in the presence of ammonium. Common regulatory mechanisms, such as accumulation of substrate and negative feedback inhibition by downstream products of nitrogen assimilation, may have affected the transcriptional and other regulatory responses to NO in a manner similar to that expected from nitrate.

A better understanding of factors that stimulate the formation of algal blooms will improve management of coastal ecosystems and inform the development of HAB prevention and mitigation strategies. The capacity to use NO as an alternate nitrogen source may provide *H. akashiwo* with an advantage over other species in areas with high concentrations of NO, such as coastal environments, or when other nitrogen sources are scarce. This study suggests that NO availability should be considered a factor in promoting toxic blooms of *H. akashiwo*.

## Methods

*H. akashiwo* was acclimated to growth on nitrate, ammonium, or a 50/50 mix of nitrate and ammonium in continuous cultures. Subcultures of each biological replicate were then spiked with NO and evaluated for gene expression, nitrate reductase enzyme activity and the uptake and assimilation of NO into biomass.

### Chemicals

^15^N-labeled S-nitroso-N-acetylpenicillamine (^15^N-SNAP, Berry & Associates/Icon Isotopes, Dexter, MI, US) was used to generate ^15^N-labeled nitric oxide. ^15^N-labelled S-nitroso-N-acetylpenicillamine (SNAP) is a donor molecule that generates ^15^N-nitric oxide^47^. There is 60% efficiency of conversion between ^15^N-SNAP and ^15^N-nitric oxide^47^. ^15^N-ammonium and ^15^N-nitrate were sourced from ^15^N-labeled ammonium chloride (Cat. 299251-1G, Sigma Aldrich, St. Louis, MO, US) and ^15^N-labeled potassium nitrate (Cat. 335134-1G Sigma Aldrich, St. Louis, MO, US).

### Algal stock conditions

Stock cultures of *Heterosigma akashiwo* (National Center for Marine Algae and Microbiota, CCMP2393) were maintained in modified Enriched Seawater/Artificial Seawater (ESAW)^48,49^. Modifications to the ESAW included an adjusted salinity of 20 PSU, and the elimination of strontium chloride hexahydrate (SrCl_2_ 6H_2_O) and sodium metasilicate nonahydrate (Na_2_SiO_3_ 9H_2_O). The nitrogen source was varied depending on experiment (see Experimental Design). Cultures were incubated at 25 °C under a 12:12 h light: dark cycle with 130 μmol m^−2^ s^−1^ irradiance.

### Continuous cultures

Continuous cultures (N = 4) were established from stock cultures acclimated to nitrate: ammonium concentrations for each experiment described below. Modified ESAW medium influx/outflux was set to maintain a cellular density of 100,000 cells mL^−1^ in 1.6 L volumes, with gentle stirring to maintain homogeneity. Cultures were incubated at 25 °C under a 12:12 h light: dark cycle with 130 μmol m^−2^ s^−1^ irradiance.

### Experimental design

Three experiments were conducted with different nitrate to ammonium ratios, while keeping total nitrogen concentrations constant (Fig. 1). Experiment 1 cultures were grown in modified ESAW with 100 µM nitrate. Experiment 2 cultures were grown in modified ESAW with 50 µM nitrate and 50 µM ammonium. Experiment 3 cultures were grown in modified ESAW without nitrate and with 100 µM ammonium. Cultures were dosed with Kanamycin (50 µg/mL) to prevent uptake and incorporation of ^15^N-labelled substrate by bacteria^50^. Subcultures were created at 4 h after the start of the light cycle resulting in 4 replicates of 4 treatments for a total of 16 subcultures.

To verify responses to more commonly encountered nitrogen sources, the treatment subcultures were spiked with ^15^N-labeled nitrogen equal to 10% of ambient nitrogen source. A fourth subculture was treated with ^15^N-labeled SNAP at a concentration equal to total nitrogen concentrations of the continuous cultures (Table 1). Subcultures were incubated at 25 °C under a 12:12 h light: dark cycle with 130 μmol m^−2^ s^−1^ irradiance.

**Table 1:** Ambient nitrogen source for continuous cultures and ^15^N-nitrogen treatment spikes for each subculture in Experiment 1, 2 and 3. SNAP, S-nitroso-N-acetylpenicillamine.

Experiment	Ambient nitrogen source	Treatment label	^15^N-labeled Nitrogen Spike
Experiment 1	100 µM nitrate	Control	ESAW media (no ^15^N)
		^15^N-nitric oxide	100 µM ^15^N-SNAP
		^15^N-nitrate	10 µM ^15^N-nitrate
		^15^N-ammonium	1 µM ^15^N-ammonium
Experiment 2	50 µM nitrate and 50 µM ammonium	Control	ESAW media (no ^15^N)
		^15^N-nitric oxide	100 µM ^15^N-SNAP
		^15^N-nitrate	5 µM ^15^N-nitrate
		^15^N-ammonium	5 µM ^15^N-ammonium
Experiment 3	100 µM ammonium	Control	ESAW media (no ^15^N)
		^15^N-nitric oxide	100 µM ^15^N-SNAP
		^15^N-nitrate	1 µM ^15^N-nitrate
		^15^N-ammonium	10 µM ^15^N-ammonium

### Gene expression (RT-qPCR)

Samples were collected for gene expression analysis using vacuum filtration onto 3 µm polycarbonate filters at 15 min and 60 min after spiking. Filters were immediately placed into RLT buffer from RNeasy Mini Kit (Qiagen, LLC Germantown, MD, US) and stored at − 80 °C until extraction. RNA was extracted using the RNeasy Mini Kit (Qiagen), treated with DNase I Amplification Grade (Thermo Fisher Scientific, Waltham, MA) as described in Coyne and Cary (2005), and reverse transcribed with random hexamers using SuperScript III First Strand Synthesis SuperMix Kit (Thermo Fisher Scientific). The resulting cDNA and a No-RT control reaction were diluted in LoTE [3 mM Tris–HCl (pH 7.5), 0.2 mM EDTA]. PCR was performed to verify reverse transcription and lack of contaminating DNA using 1 μL diluted template, 0.2 mM dNTPs, 2.5 mM MgCl_2_, 1X Taq polymerase buffer (Sigma Aldrich, St. Louis, MO, US), 0.25 units Jump-Start Taq Polymerase (Sigma Aldrich), 1 μg BSA, and 3 μM of GAP primers (Table 2) in a 20 μL reaction volume. Reactions proceeded for 35 cycles of 94 °C for 30 s, 56 °C for 30 s, and 72 °C for 1 min.

**Table 2:** Primer sequences and concentrations for qPCR.

ID (Reference)	Primers	Sequence (3′–5′)	Primer concentration
GAP (Coyne^28^)	HaGAP-448F	CAAGTGCATTGATGATGCCTT	3 µM
	HaGAP-638R	GTCAGCTTACCCTTCAGGT	3 µM
NR (Coyne^28^)	HaNR-F	TCCCTGGGCAGAAAGCAACT	3 µM
	HaNR-R	GCATGATGGGCACCTCATACT	3 µM
GS	Ha-GS-286-F	TCGATGGTTCTTCCACTGGC	0.5 µM
	Ha-GS-570-R	GAGGGTAACCAGACTTGGGC	0.5 µM
GOGAT	Ha-GOGAT-80F	GTGGTGTTGGTTTCATCGCC	9 µM
	Ha-GOGAT-261-R	GCCGTTGGTGTACTCCTCAA	0.5 µM

Primers for the cytosolic form of glutamine synthetase (*GS*) and glutamate synthase (*GOGAT*) were designed using the “Primer-BLAST” function in Basic Local Alignment Search Tool (BLAST)^51^ from National Center for Biotechnology Information (NCBI). Primers were designed from sequences in the 20130911 combined assembly for *H. akashiwo* CCMP2393 transcriptome, downloaded from the Moore Foundation Marine Microbial Eukaryote Transcriptome Sequencing Program^52^. PCR was conducted on cDNA with each primer pair and analyzed for the correct size fragment using gel electrophoresis. Primers were then optimized by qPCR and dissociation curves were evaluated to confirm specificity of primers.

Transcript abundance was measured by qPCR for *GAP*, *NR*, *GS* and *GOGAT* using an ABI 7500 Real-Time PCR System (Thermo Fisher Scientific). Diluted cDNA was used as template in triplicate 10 µL reactions. Each reaction consisted of 1 µL of template cDNA, 5 µL of SYBR Select Master Mix (Thermo Fisher Scientific), 2 µL sterile water, 1 µL each of forward and reverse primers (Table 2). qPCR cycling parameters were as follows: 50 °C for 2 min and 95 °C for 10 min, followed by 40 cycles of 95 °C for 15 s, 60 °C for 30 s and 72 °C for 1 min. A dissociation stage was added to assess the specificity of amplification. Average transcript abundance was evaluated by linear regression analysis of triplicate reactions. Transcript abundance for *NR*, *GS* and *GOGAT* was then normalized to *GAP*^9,28^.

### Nitrate reductase activity

Samples were collected from subcultures by centrifugation at two hours after spiking. The supernatant was discarded and the cell pellet was immediately frozen in liquid nitrogen. Samples were stored at − 80 °C until analysis. The frozen cell pellets were resuspended in 200 mM KPi extraction buffer (27.2 g L^−1^ KH_2_PO_4_, 10 g L^−1^ KOH, adjusted to pH 7.9) on ice. The resuspended pellet was homogenized by sonication, and centrifuged to clarify at 4 °C. The supernatants were immediately assayed as follows: triplicate 100 µL aliquots of extract were each mixed with 100 µL of 500 mM KPi assay buffer (pH 7.9) and 25 µL of 2 mM NADH (Sigma-Aldrich). KNO_3_ (100 µM) was added to duplicate assays and sterile water was added to the third as a negative control reaction in place of KNO_3_. The reaction was incubated at room temperature for 15 min and 1 M zinc acetate was added to stop the reaction. The samples were centrifuged to pellet the zinc acetate and the supernatant was transferred to a new tube. The concentration of nitrite in the supernatant was measured colorimetrically by addition of 698 µM N-methylphenozonium methyl sulfate (Sigma-Aldrich). After incubation at room temperature for 20 min in the dark, 5 M HCl and 0.058 M sulfanilamide (Sigma-Aldrich) was added and the reaction was incubated at room temperature for another 5 min in the dark. 0.004 M N-(1-Naphthyl) ethylenediamine hydrochloride (Sigma-Aldrich) was then added and the reaction was incubated at room temperature for 10 min in the dark. Absorbance was measured at 543 nm using a Thermo Scientific Nanodrop 2000c (Thermo Fisher Scientific). NR activity was normalized to protein content of the cellular homogenate as determined using the Pierce BCA Protein Assay Kit (Pierce, Rockford, IL, US).

### Nitrogen assimilation (^15^N)

Samples were collected at 4 and 24 h after spiking for particulate ^15^N analysis. Subsamples of each culture were vacuum filtered onto GF/F filters and dried at 60 °C for 24 h. Filters were folded into combusted tins and pelletized. Particulate ^15^N in pelletized samples was measured by mass spectroscopy at Boston University (Experiment 1) or UC Davis (Experiments 2 and 3). Nitrogen uptake per biomass (V_m_, per hour) was calculated according to methods outlined in Dugdale and Wilkerson^53^ and the uptake rate of nitrogen (Rho, µM/hr) was calculated according to Middelburg and Nieuwenhuize^54^ and Dugdale and Wilkerson^53^.

### Statistical analysis

Statistical analyses were performed in R using base functions and the “car” package. Gene expression and NR assay data were first evaluated using the Q Test^55^ and outliers (identified at 95% confidence level) were removed. A Shapiro test on the residuals from a linear model was used to assess normality. If the *p* value was not significant (*p* value > 0.05), we assumed normality. A Levene test was used to assess homogeneity of variance. If the *p* value is not significant (*p* value > 0.05), we assumed homogeneity of variance.

A one-way ANOVA with a post-hoc Tukey’s test was employed when the data were normal, and variance was homogeneous. A paired t-test was used when the data were normal, but not homogeneous. *P* values from the paired t-test were adjusted using the Benjamini and Hochberg Correction^56,57^. Data that were not normally distributed were log transformed. Significance was determined using one-way ANOVA analysis. If normality could not be corrected using a log transformation, a Kruskal Wallis and a post-hoc Pairwise Wilcox Sign Test was used. Statistically significant differences were defined by a *p* value < 0.05.

## Supplementary Information

Supplementary Information.

## Data Availability

The data generated during the current study are available in the following dataset: https://doi.org/10.5281/zenodo.7293238.
