# Supplementary material for: Effects of nitrate and ammonium on assimilation of nitric oxide by Heterosigma akashiwo
Source: Sci Rep. 2023 Jan 12;13:621. doi: 10.1038/s41598-023-27692-3 (PMC9837059; doi:10.1038/s41598-023-27692-3)
Supplement: Supplementary file 1 — Supplementary Information. [file 41598_2023_27692_MOESM1_ESM.docx]

Supplementary Figures and Calculations

**Effects of nitrate and ammonium on assimilation of nitric oxide by *Heterosigma akashiwo***

Emily M. Healey, Stacie Flood, Patience Bock, Robinson W. Fulweiler, Joanna York, Kathryn J. Coyne

Supplementary Figure S1


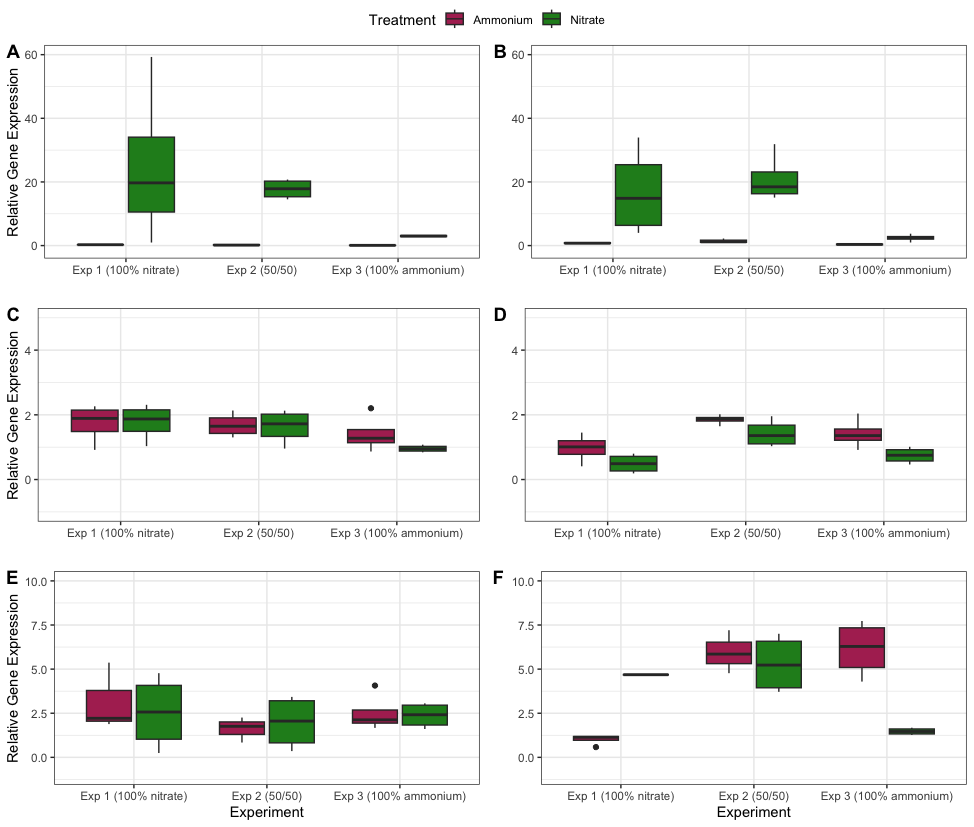


Relative gene expression of nitrate reductase (*NR*; A, B), glutamine synthetase (*GS*; C, D) and glutamine:2-oxoglutarate aminotransferase (*GOGAT*; E, F) at 15 minutes (A, C, E) and at 60 minutes (B, D, F) after spiking with nitrate or ammonium for cultures of *Heterosigma akashiwo* when acclimated to growth on 100 µM nitrate (Exp 1), 50 µM nitrate and 50 µM ammonium (Exp 2), or 100 µM ammonium (Exp 3). Expression was normalized to *GAP* transcript abundance. Error bars are +/- 1 standard deviation from the mean.

Supplementary Figure S2


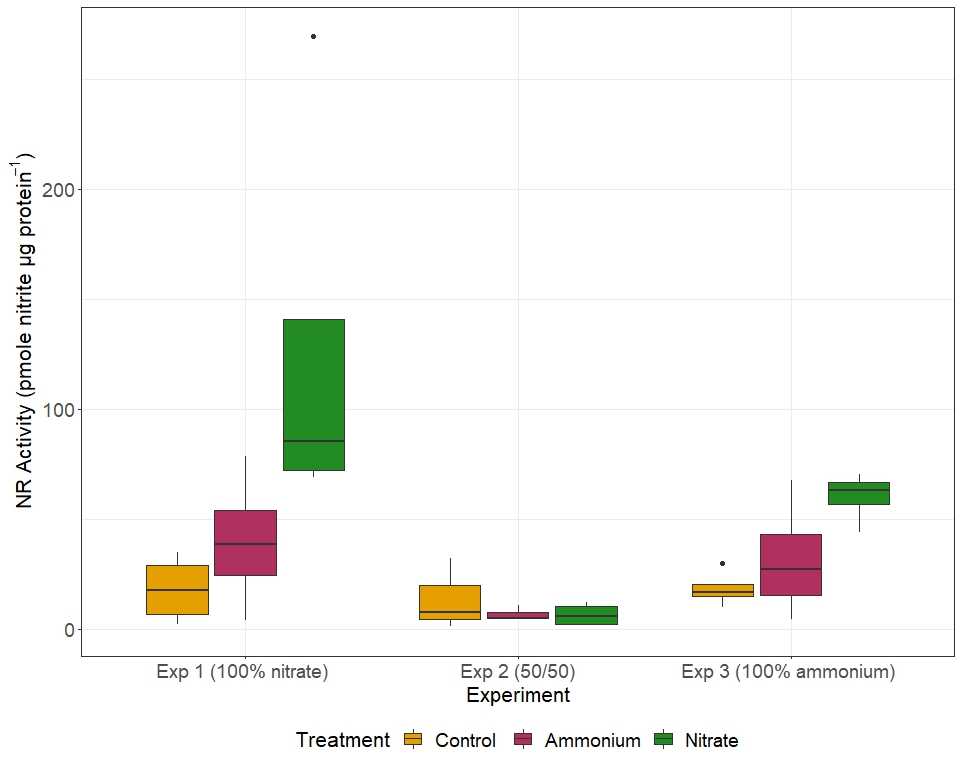


Nitrate reductase (NR) enzyme activity 2 hours after spiking with nitrate or ammonium for *Heterosigma akashiwo* cultures when acclimated to growth on 100 µM nitrate (Exp 1), 50 µM nitrate and 50 µM ammonium (Exp 2), or 100 µM ammonium (Exp 3). Expression was normalized to protein content. Error bars are +/- 1 standard deviation from the mean.

**Supplemental Calculations**

The amount of nitrogen uptake per biomass (Vm, per hour) were calculated according to methods outlined in Dugdale and Wilkerson (1986):

Vm = (Vo + Vt) / 2

Vo = 15Nxs / ([15Nenr – {F}] x T)

Vt = 15Nxs / ([15Nenr – 15Ns] x T)

where 15Nxs is the atom percentage excess in sample, 15Ns is the atom percentage of 15N in the initially labeled fraction, 15Ns is the atom percentage of 15N in sample. T is the incubation time, and {F} is the naturally occurring abundance of 15N. V was multiplied by particulate nitrogen concentration to calculate the uptake rate of nitrogen (Rho, μM/hr) (Middelburg and Nieuwenhuize 2000; Dugdale and Wilkerson 1986):

Rho = Vm x PON

Dugdale, R. C., & Wilkerson, F. P. (1986). The use of 15N to measure nitrogen uptake in eutrophic oceans; experimental considerations 1, 2. *Limnology and Oceanography*, 31(4), 673-689.

Middelburg, J., & Nieuwenhuize, J. (2000). Nitrogen uptake by heterotrophic bacteria and phytoplankton in the nitrate-rich Thames estuary. *Marine Ecology Progress Series*, 203, 13–21.
